# Real‐Time Internal Steam Pop Detection during Radiofrequency Ablation with a Radiofrequency Ablation Needle Integrated with a Temperature and Pressure Sensor: Preclinical and Clinical Pilot Tests

**DOI:** 10.1002/advs.202100725

**Published:** 2021-08-05

**Authors:** Jaeho Park, Dong Ik Cha, Yongrok Jeong, Hayan Park, Jinwoo Lee, Tae Wook Kang, Hyo Keun Lim, Inkyu Park

**Affiliations:** ^1^ Department of Mechanical Engineering Korea Advanced Institute of Science and Technology (KAIST) Daejeon 34141 South Korea; ^2^ Radiology and Center for Imaging Science Samsung Medical Center Sungkyunkwan University School of Medicine Seoul 06351 South Korea; ^3^ RF Medical Co. Ltd. Seoul 08511 South Korea; ^4^ Department of Health Sciences and Technology Samsung Advanced Institute for Health Sciences & Technology (SAIHST) Sungkyunkwan University School of Medicine Seoul 06355 South Korea; ^5^ Present address: Department of Chemical Engineering Stanford University Stanford CA 94305 United States

**Keywords:** flexible sensors, radiofrequency ablation, sensor integrated medical needles, steam pop, temperature and pressure sensors

## Abstract

A radiofrequency ablation (RFA) needle integrated with a temperature sensor (T‐sensor) and pressure sensor (P‐sensor) is designed and utilized for real‐time internal steam pop monitoring during RFA. The characteristics of the sensor‐integrated RFA needle (sRFA‐needle) are investigated quantitatively using a pressure chamber system, and the feasibility and usability of the needle in preclinical and clinical trials is demonstrated. The sharp changes in the temperature and normalized pressure sensor signals induced by the abrupt release of hot and high‐pressure steam can be clearly monitored during the steam pop phenomena. The basic mechanism of the preliminary steam pop is hypothesized and verified using in situ ultrasound imaging data and computational analysis data of the RFA procedure. Moreover, the usability of the system in clinical trials is investigated, and the steam pop phenomena during the RFA procedure are detected using T‐sensor and P‐sensor. The results confirm that the sensor integration on the medical needle can provide critical data for safer and more effective medical practices.

## Graphical abstract

A radiofrequency ablation (RFA) needle integrated with a flexible thin‐film type temperature and a pressure sensor is reported and its clinical applications toward real‐time detection of internal steam pop and monitoring of environmental change in the ablation zone are studied. The study is performed in a pre‐clinical trial (animal study) and clinical trial (human study) to verify their usefulness in an in‐vivo environment.

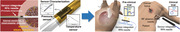

## 1 Introduction

**Figure 1:**
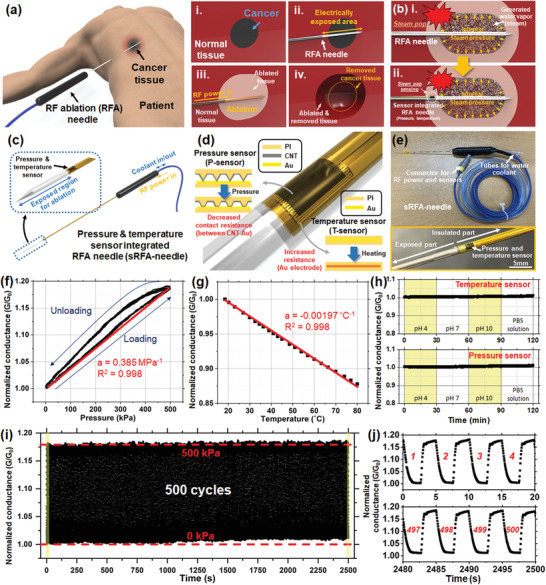
Schematic of RFA procedure and sRFA‐needle and characteristics of the sensor on the RFA needle. a) Schematic of the RFA procedure to remove cancer tissue in patients: i) cancer in the normal tissue; ii) insertion of RFA needle through cancerous tissue; iii) application of RF power to the RFA needle and ablation of the tissue due to the increased temperature; iv) removal of cancerous tissue after the RFA procedure. b) Schematic of the steam pop phenomena and proposed sRFA‐needle to detect and monitor steam pop. c) Schematic of the overall system for the sRFA‐needle. d) Schematic of the working principles of contact resistance‐based P‐sensor and resistance‐based T‐sensor. e) Photograph of sRFA‐needle and magnified image (inset) of sensing point. Relative conductance change of f) P‐sensor under hydrostatic air pressure and g) T‐sensor under increasing surrounding temperature. h) Change in normalized conductance of sensor under various chemical environments, including different pH solutions and a phosphate‐buffered saline solution. i) Change in the relative conductance of the P‐sensor over 500 cycles of cyclic hydrostatic pressure loading, and j) profile of relative conductance during the first and last four cycles.

Radiofrequency ablation (RFA) is a minimally invasive surgery technique to remove a problematic parts of tissues for medical treatment and has been widely applied to treat cancers and cardiovascular diseases.^[^ ^1^, ^2^, ^3^, ^4^ ^]^ In this procedure, as shown in **Figure** **1**a, an alternating current with a frequency of ≈200–400 kHz is injected through the RFA needle, which is placed near the tissue to be removed; then, the ionic agitation of electrolytes inside the tissue occurs.^[^ ^4^, ^5^ ^]^ This agitation generates heat and increases the temperature of the tissue up to 50–100 °C. As a consequence, the tissue near the RFA needle is thermally ablated and undergoes hyperthermia‐induced coagulation necrosis. During the RFA procedure, an unintended audible explosion called “steam pop” occurs, due to the increased internal steam pressure in the ablation region, as described in Figure 1b. It has been pointed out that steam pop during the RFA procedure can induce various thermal and mechanical effects on neighboring tissues. In the case of cancer RFA, the relationship between steam pop and cancer recurrence is still controversial.^[^ ^6^, ^7^, ^8^, ^9^, ^10^ ^]^ Some researchers reported that steam pop is not related to the probability of early local tumor progression,^[^ ^8^, ^9^ ^]^ while others reported that steam pop may cause tumor recurrence.^[^ ^6^, ^7^, ^10^ ^]^ On the other hand, the results of Fernandes et al.^[^ ^8^ ^]^ implied that steam pop could be negatively associated with tumor recurrence. In the case of cardiovascular disease, it has been reported that steam pop can cause perforation during the RFA procedure, which can be a significant danger to the patient.^[^ ^11^, ^12^ ^]^ However, methods for the detection and monitoring of local environmental changes (e.g., temperature and pressure) near the needle tip during the RFA procedure have not been established. Some studies reported the monitoring of the environment in the ablation zone by utilizing measurable parameters from the RFA needle, such as radiofrequency (RF) power,^[^ ^13^, ^14^ ^]^ electrical impedance,^[^ ^15^, ^16^, ^17^, ^18^ ^]^ temperature,^[^ ^16^ ^]^ and others.^[^ ^19^ ^]^ However, for a more direct measurement of the physical conditions of the tissue during RFA, miniaturized sensors integrated on the needle tip surface should be developed. Conventional microelectromechanical system (MEMS)‐based sensors are suitable for this purpose as they are widely used in catheter systems. However, because the RFA needle contains various functional components and has very small dimensions (diameter <1.5 mm) compared to the catheter, the integration of the MEMS sensor is extremely difficult. As an alternative solution, the RFA needle has been integrated with an optical‐fiber‐based temperature or pressure sensor;^[^ ^19^, ^20^, ^21^ ^]^ however, this approach is only applicable to needles with larger diameters (≈3 mm) owing to the complexity of the assembly process of the optical sensing components within the RFA needle. Furthermore, various methods in both noninvasive and invasive ways to predict the temperature during thermal treatment have been studied,^[^ ^22^, ^23^ ^]^ but multiparametric observation by integrated multiple sensors into the RFA needle was not still fully studied. Recently, our group reported multiplexed microscale sensor devices on a thin, flexible polymer platform with the capability of sensing various biophysical and biochemical properties, including the pressure, temperature, electrical impedance, pH, glucose, and lactate concentration.^[^ ^24^, ^25^, ^26^ ^]^ These flexible microscale sensors could be integrated onto the surface of medical needles with a diameter of less than 1.5 mm, which is difficult in the case of wire and fiber‐based assembly for sensor fabrication.^[^ ^19^, ^20^ ^]^ In a previous study, we integrated flexible thin film pressure sensor (P‐sensor) and temperature sensor (T‐sensor) on an RFA needle to detect the steam pop during the RFA procedure in an ex vivo environment.^[^ ^26^ ^]^ However, this only provided a proof‐of‐concept study of the sensor‐integrated RFA needle (sRFA‐needle) as a method for steam pop detection. Hence, here, we report further progress of the sRFA‐needle toward real clinical applications through a quantitative analysis of the sensor characteristics from the system point of view (i.e., the sRFA‐needle) within a pressure‐controlled vessel, preclinical tests with animal models, and clinical pilot tests with human patients. The feasibility and usefulness of the sRFA‐needle system were demonstrated in both preclinical and clinical trials.

## 2 Results and Discussion

### 2.1 System and Sensor Characteristics of the sRFA‐Needle

Figure 1c shows a schematic of the sRFA‐needle. The RFA needle contains an internal tube and a thermocouple to maintain the temperature as low as possible during the RFA process by circulating the water coolant through the tube. The RF power for ablation is provided through the metal needle body, and the electrical power is transmitted to the tissue through the exposed part (≈3 cm) of the metal needle, which is not encapsulated by the polymeric insulation tube. A flexible sensor based on a polymeric platform with pressure and temperature sensing capabilities is integrated on the surface of the RFA needle, and the sensing point is placed at the end of the insulated part of the needle, as displayed in Figure 1c. The sensing mechanisms of the P‐sensor and T‐sensors are shown in Figure 1d. For the P‐sensor, the change in contact resistance between the metal electrodes and carbon nanotubes (CNTs) on the surface of the microstructured polyimide substrate (CNT‐PI) was measured and utilized for pressure sensing. On the other hand, the T‐sensor is a resistance temperature detector, which words based on the principle that the electrical resistance of a metal electrode is linearly dependent on the temperature. As shown in Figure 1e, the sensor was conformally assembled on the surface of a needle with a diameter of 1.5 mm and length of 15 cm, and the customized handle packaging was fully assembled for enhanced usability. The characteristics of the sensors on the needle were fully characterized in a pressure vessel customized for the experiment, and the hydrostatic pressure was precisely applied to the sensor (see the Supporting Information for the details of the customized pressure vessel). Figure 1f shows the relative conductance change of the P‐sensor under air pressure increasing and decreasing in the range of 0–500 kPa (increasing/decreasing rate = 12.5 kPa s^−1^). Although there is a slight hysteresis (13.13%), the P‐sensor shows an almost linear response to the elevating pressure with a sensitivity, which is defined as the ratio of the change in relative conductance (Δ*G*/*G*_0_) to the change in pressure (Δ*P*), of 0.385 MPa^−1^ (*R* ^2^ = 0.998). The sensing mechanism was based on the contact resistance change due to the applied pressure, and it could be confirmed from the linear response between the logarithmic scale of response and pressure (Figure S2, Supporting Information for the detailed explanation of the P‐sensor behavior based on the contact theory). In the case of the T‐sensor, as shown in Figure 1g, the relative change in conductance decreased linearly with increasing temperature with a sensitivity of −0.00197 °C^−1^ (*R* ^2^ = 0.998). Because the RFA needle is usually placed in an environment with various chemical components, the influence of the environment on the sensor signal is critical. Therefore, the stability of the P‐sensor and T‐sensor in the sRFA‐needle was investigated not only in the physiological environment but also in extreme cases such as acidic and basic environments. As shown in Figure 1h, the sRFA‐needle was sequentially placed in pH buffer solutions with pH 4, 7, and 10, and a conductive physiological phosphate‐buffered saline solution, and the sensors showed stable signals with almost negligible changes in the relative conductance. Furthermore, upon 500 cycles of pressure loading, the sRFA‐needle did not show any significant drift or change in response, as shown in Figure 1i–j, which verifies the stability of the sRFA‐needle system. It should be noted that the temperature could affect the conductance of the P‐sensor (Figure S3a,b, Supporting Information for more details on the temperature effect on the P‐sensor). However, as shown in Figure S3c in the Supporting Information, the temperature effect on the sensor could also be compensated by utilizing the in situ temperature measurement system.

### 2.2 Computational Analysis for the Early Progression of the Ablation Region and the Steam Pop Generation

**Figure 2:**
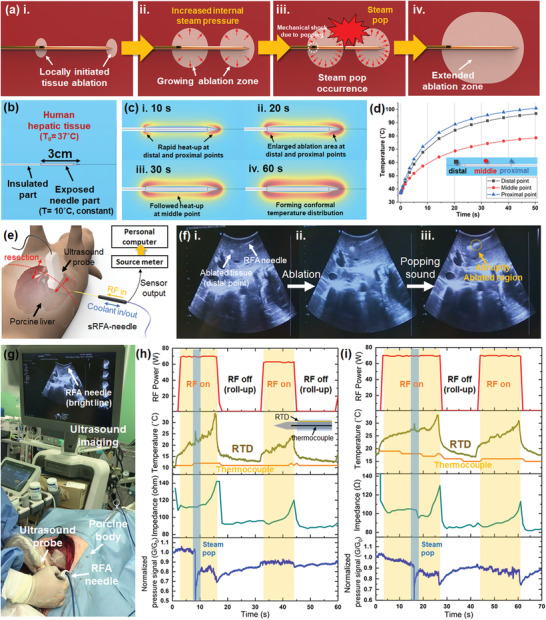
Mechanism of preliminary steam pop during RFA procedure and preclinical trials of the sRFA‐needle with the pig. a) Schematic of the hypothesized mechanism of the steam pop in the preliminary stage: i) local initiation of the ablation at the exposed region (i.e., distal and proximal points of the needle); ii) growth of ablation zone and increase in internal steam pressure due to high temperature; iii) occurrence of the steam pop due to increased steam pressure at the distal and proximal points of the needle; iv) extended ablation zone after steam pop. b) Model of the computational analysis to calculate the distribution of temperature around the RFA needle, and c) results of the analysis: i) 10 s after ablation with rapid heat‐up at both distal and proximal points; ii) 20 s after ablation with an enlarged ablation zone; iii,iv) 30 and 60 s after ablation, respectively, with followed heat‐up at the middle point of the needle; d) numerically calculated temperature profile at the distal, middle, and proximal points of the RFA needle during ablation. e) Schematic of preclinical trials: the abdomen of the pig was resected and the sRFA‐needle was inserted to the porcine liver while the ablation was monitored through the ultrasound imaging probe. f) Consecutive snapshots of ultrasound imaging during the RFA procedure: i) early stage of the RFA procedure (the ablated tissue at the distal point of the needle can be clearly seen); ii) extended ablated tissue at the distal point of the needle; iii) ultrasound image immediately after the occurrence of steam pop (the ablation zone was abruptly extended in the middle point of the RFA needle). g) Photograph of the experimental setup for the preclinical trial. h,i) Results of measured RF power, temperature, impedance, and normalized pressure signal during the RFA procedure.

Although steam pop is induced by the increased internal steam pressure in the tissue due to increases in temperature, generalizing the tendency of steam pop is almost impossible because it significantly depends on various factors, such as the structure of the surrounding tissue, position of the needle, applied RF power, and temperature of the cooled needle tip. However, we could hypothesize that primary steam pop is usually induced by the rapid increase in temperature at the distal and proximal points of the exposed needle, as schematically illustrated in **Figure** **2**a. When RF power is applied to the RFA needle, the current density at the distal point (the needle tip) and the proximal point (the boundary between the exposed and insulated needle) is significantly higher than that at the middle point of the ablation zone. Therefore, the tissues are preferentially ablated at these locations, while water in the tissue is vaporized and the internal steam pressure increases. Therefore, when the tissue at the middle point starts to be ablated, increased internal pressure at both the distal and proximal points could cause explosions toward the middle point, producing audible popping sounds. When steam pop occurs, the released hot steam can extend the ablation zone because the spread hot steam could rapidly increase the tissue temperature. To confirm this hypothesis, the RFA procedure of the human liver was numerically simulated, and the temperature around the RFA needle with a needle exposure of 3 cm was estimated (Figure 2b–d). The temperature of the RFA needle was fixed (10 °C) because the water coolant was circulated to maintain the temperature of the needle body as low as possible during the RFA procedure. As illustrated in Figure 2ci,d, the temperatures at the distal and proximal points are increased rapidly, and a large amount of tissue reaches a temperature of ≈75 °C within 10 s, while most of the tissue at the middle point remains below 60 °C. This imbalance in the temperature between the edge and the middle point of the RFA needle becomes severe, and temperatures at the distal and proximal points increase up to 90 °C in a few tens of seconds, indicating the vaporization of water in the tissue and the induction of internal steam pressure.

### 2.3 Preclinical Trials of the sRFA‐Needle in Porcine Samples under Ultrasound Imaging

The feasibility and usability of the sRFA‐needle were confirmed by a preclinical study on pig samples. Figure 2e,g shows a schematic and a photograph of the experimental setup. The sRFA‐needle was inserted into the porcine liver of a live pig. The ablation process inside the liver tissue was monitored using an ultrasound probe. The ground pad was attached to the other part of the pigskin, and an RF power of 70 W was supplied to the RFA needle to start the tissue ablation. Figure 2f shows snapshot ultrasound images captured during the RFA process. From this figure, it can be seen that the ablation zone was clearly visible at the distal point of the RFA needle. In general, when the temperature around the RFA needle increases due to Joule heating, vaporization occurs and it generates steam inside the tissue. Because the acoustic impedance of generated steam is different from that of the biological tissue, the ablated region becomes echogenic on ultrasound due to vaporized air within the ablation zone. However, when the ablation further proceeded, an audible popping sound was detected and an abrupt progression of tissue ablation was observed in the ultrasound image (Figure 2fiii). This trend of steam pop generation at the initial stage of the RFA procedure could also be detected in other RFA processes (Video S1 in the Supporting Information of the ultrasound imaging during RFA procedure). Thus, we could confirm that the steam pop phenomena generally occur owing to the imbalance in the temperature near the RFA needle and the release of hot steam accumulated at the distal and proximal points. The measured RF power, temperature, electrical impedance, and normalized P‐sensor signals are presented in Figure 2h,i. When steam pop occurred during the RFA procedure, clear spike signals in the T‐sensor and P‐sensor could be detected. The measurement results indicate that steam pop involves a sudden release of internal steam with high pressure, and the accompanying thermal and mechanical shock can change the signals of the T‐sensor and P‐sensor of the sRFA‐needle. The measured normalized pressure signal, which is the relative conductance change, significantly decreased during steam pop, and was restored immediately after popping. This result indicates that the initial pressure applied to the P‐sensor by the surrounding tissue is suddenly removed and the hot steam expands the gap between the tissue and the P‐sensor, which may float the CNT‐PI layer from the Au sensor electrode consequently raising the contact resistance (Figure 1d). However, after the abrupt popping, the CNT‐PI layer recovers its electrical contact with the Au sensor electrodes, as observed by the restored signal of the P‐sensor. An abrupt increase in temperature could be observed due to the hot steam during the above‐mentioned explosion step. However, the electrical impedance, which is the representative measurable parameter in conventional RFA procedures, could not precisely reflect the occurrence of steam pop, as shown in Figure 2h,i. Thus, the sRFA‐needle could immediately capture and monitor the occurrence of steam pop, providing insight about the environmental changes near the RFA needle during the RFA procedure, which can be utilized for further scientific and clinical analysis of the RFA procedure. Furthermore, the ablation volume of the sRFA‐needle was found to be identical to that of the conventional cool‐tip RFA needles without integrated sensors, as described in Figure S4 in the Supporting Information.

### 2.4 Human Clinical Trials of the sRFA‐Needle

**Figure 3:**
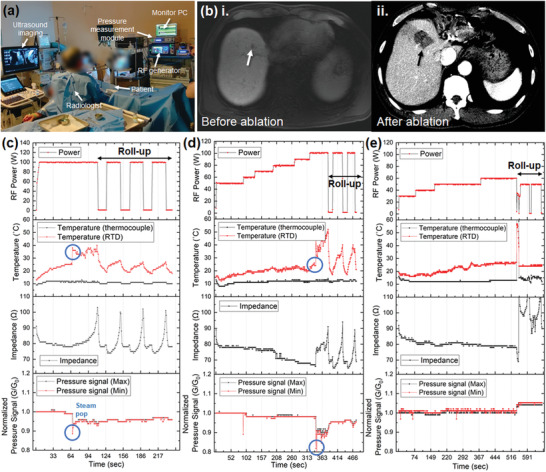
Clinical trials of the RFA procedure with the sRFA‐needle. a) Photograph of the clinical setup, and b) computed tomography image of the patient before and after the procedure: i) before the RFA procedure, where a nodule of a hepatocellular carcinoma (white arrow) is clearly visible in the image; ii) after the RFA procedure; the liver tissue around the hepatocellular carcinoma nodule (black arrow) was ablated and removed. c–e) Monitored parameters (temperature, impedance, and normalized pressure signal) during the RFA procedures for different patients. c,d) Steam pop during the RFA procedure; the corresponding peak and change in the temperature and pressure signals can be clearly observed, while e) no significant peak was observed in a patient during the RFA procedure owing to the absence of the steam pop phenomena.

The applicability of the proposed system was further investigated in real clinical trials with human patients. **Figure** **3**a shows a photograph of the setup for clinical trials. The measurement equipment used in this study for the T‐sensor and P‐sensor was certified by the Korea Testing Certification Institute (KTC) and the Ministry of Food and Drug Safety (MFDS) in Korea in terms of electrical, biological, mechanical, and electromagnetic interference safety. This equipment was utilized owing to safety issues during the clinical trials. Figure 3b shows computed tomography (CT) images of the patient corresponding to the in situ measurement results in Figure 3d before and after the RFA procedure. As shown in Figure 3bi, the small nodule of hepatocellular carcinoma (HCC) indicated by the white arrow in the arterial phase image, was successfully ablated as indicated by the black arrow in Figure 3bii. For the clinical trials, the RFA procedures with the sRFA‐needles were performed for ten patients (see the Supporting Information for details on the patient information of the clinical trial). Figure 3c–e is the representative in situ measurement results of the RFA procedures with the sRFA‐needles. As shown in Figure 3e, the two cases did not involve the steam pop phenomena during the RFA procedure. However, in six other cases, abrupt changes were observed in the T‐sensor and P‐sensor signal when steam pop occurred, as presented in Figure 3c,d. As described in the preclinical test, the measured temperature value increased owing to the sudden spurt of hot steam during the steam pop, and the P‐sensor signal abruptly decreased because the sudden release of steam at high pressure could expand the gap between the CNT‐PI layer and the Au electrodes. The differences of the calculated relative P‐sensor signal were 0.105 and 0.164 in Figure 3c,d, respectively (see the Supporting Information for the summary of measurable parameters during the RFA treatment in clinical trials) During the steam pop, a radiologist, who performed the RFA procedure, clearly heard the explosive shock to the RFA needle and observed the peak of the P‐sensor signal simultaneously, which further verifies that the sensor on the RFA needle could detect the steam pop. The temperature differences at T‐sensor between before and after of steam pop were about 13 and 21 °C, while those at thermocouple inside the RFA needle were about 1 and 0 °C in Figure 3c,d, respectively. It clearly shows that the location of the sensor is crucial for the temperature monitoring during the RFA procedure, and placing the temperature sensor at the outer surface rather than inside of the RFA needle can readily detect and monitor the temperature changes in the tissue around the RFA needle. In the case of electrical impedance, the measured value decreased after the steam pop in Figure 3c. However, in Figure 3d, no change was observed despite the steam pop, which means that the electrical impedance is not the appropriate parameter to detect the steam pop or to monitor environmental change around the RFA needle. In some case, as shown in Figure S5 in the Supporting Information, the sensor could not detect the steam pop despite the fact that the radiologist could sense the shock during the procedure. It is considered that the steam pop might have occurred at the distal point of the needle, and the released hot steam could not reach the sensor, which was placed at the proximal point (see the Supporting Information for the result of the false‐positive case). The sensor could not be integrated onto the distal point or part of the exposed needle because the sensor component was made of nonconducting materials, which can hinder the ablation performance. Thus, a new sensor structure that can detect not only the proximal point but also other locations of the needle tip, while not deteriorating the RFA performance, should be designed in the future. However, it is believed that monitoring the physical parameters at the ablation zone during the steam pop phenomenon can help the quantitative analysis of the RFA procedure and thus make it more effective and safer.

## 3 Conclusion

In this study, we integrated a thin polymeric sensor platform on an RFA needle with a diameter of 1.5 mm to monitor the environment of tissues and detect steam pop during RFA. The characteristics of the sRFA‐needle were investigated in a customized pressure chamber. The feasibility and usability of the proposed sRFA‐needle were verified in preclinical tests through pig and human clinical tests. The integrated sensors reliably detected the occurrence of steam pop, and we confirmed that the hot steam in the tissue was rapidly spread during the steam pop. Although this study could neither confirm the origin nor provide prediction of the steam pop phenomena, it provides insight into the environmental changes in tissues during RFA. It is expected that diverse properties of the tissues undergoing RFA could be characterized by utilizing various physical/chemical sensor arrays integrated on the RFA needle. Furthermore, we believe that integrating sensors on the medical needle can provide useful information about a variety of medical procedures and accompanying environmental changes in the human body, which can be further utilized for developing more effective and safer surgery procedures.

## 4 Experimental Section

### Preparation of the sRFA‐Needle

T‐sensor and P‐sensor on the polymeric platform were fabricated using a previously reported method.^[^ ^26^ ^]^ For the substrate, a polyimide (PI‐1388, VTEC, USA) varnish was spin‐coated on a bare silicon wafer followed by baking at 150 °C for 30 min and an imidization process at 250 °C for 3 h. Then, metal patterning for the formation of metal electrodes was performed using a lift‐off process, including photolithography and e‐beam evaporation. The P‐sensor was further assembled with a microstructured polyimide‐carbon nanotube (PI‐CNT) composite using a customized polyimide (thickness ≈5 µm) tape to induce a pressure‐responsive contact resistance with gold (Au) electrodes. For the integration of the sensor onto the RFA needle, a pressure‐sensitive adhesive (PSA, MD‐7, Dow Corning, USA) for the temporal adhesion of the sensor on the RFA needle and a polyethylene terephthalate (PET) heat‐shrink tube (Nordson Medical, USA) were utilized. The inner PET heat‐shrink tube was coated on the RFA needle using a heat‐gun at 150 °C, and the sensor coated with PSA on the backside was attached to the surface of the inner heat‐shrink tube. Then, additional insulation tubing with an outer PET heat‐shrink tube was formed, and the edge of the outer heat‐shrink tube was sealed with a medical‐grade UV‐curable adhesive (Loctite 3321, Henkel, Germany). Finally, an additional thermal treatment was performed to stabilize the mechanical properties of the outer PET heat‐shrink tube.

### System Characterization in Pressure Vessel

The sRFA‐needle system was characterized in a customized pressure vessel to mimic the tissue environment during the RFA procedure. The pressure in the pressure vessel was controlled using an electronic air pressure regulator (ITV1000, SMC Corp., Japan), source meter (2400, Keithley, USA), and customized LabView (National Instruments Corp., USA) program (see the Supporting Information for detail of the operation scheme of the customized pressure vessel). The resistances of both the T‐sensor and P‐sensor were measured using a two‐channel source meter (2602, Keithley, USA), and the measured data were logged in a separate text file with the corresponding pressure values in the vessel.

### Computational Analysis of the Temperature Distribution around the RFA Needle during the RFA Procedure

A computational analysis of the temperature distribution around the RFA needle was carried out using the commercial software COMSOL (COMSOL, Inc.). The model consisted of the RFA needle and the surrounding human hepatic tissue. The model of the RFA needle had both the noninsulated and insulated parts, and the length of the noninsulated part was set to 3 cm (see Figure S6 in the Supporting Information for more details about the model of computational analysis) The initial temperature of the model of human hepatic tissue was set to 37 °C, while the surface of the needle was maintained at 10 °C during the RFA procedure to mimic the cool‐tip RFA needle. To simplify the model, a DC potential of 60 V, instead of an AC potential, was applied to the body of the RFA needle while the exterior boundary of tissue was considered as electrically grounded. The effect from phase transition of the water due to vaporization was neglected in order to simplify the simulation model because the temperature distribution around the RFA needle before vaporization was concerned. Then, the generated electrical current density and heat from Joule heating were calculated. The heat measurements were further utilized to simulate the heat transfer in the model of human hepatic tissue. The heat transfer in the biological medium was calculated based on the following Penne's equation^[^ ^27^, ^28^ ^]^

(1) $$\rho c\frac{dT}{dt}=\nabla \cdot k\nabla T+Q-Q_{p}+Q_{m}$$

where *ρ*, *c*, *k*, *T*, *Q, Q*_p_, and *Q*_m_ are the mass density, specific heat capacity, thermal conductivity, temperature, generated Joule heat, heat loss due to blood perfusion in the tissue, and generated metabolic heat, respectively. The heat *Q*_p_ induced by the microvascular blood flow maintains the temperature of the tissue to the body temperature, and it can be further modeled as follows

(2) $$Q_{p}=\omega_{b}c_{b}\left(T-T_{b}\right)$$

where *ω*_b_ *, c*_b_, and *T*_b_ are the blood perfusion rate, specific heat capacity of the blood, and temperature of the blood, respectively. For the simulation, the thermal and electrical parameters of human hepatic tissue were used, including the blood perfusion rate, reported by Schutt and Haemmerich^[^ ^29^ ^]^ After the calculation for heating with 1 min of voltage injection, the temperature values at a 2 mm distance from the surface of the RFA needle at the distal, middle, and proximal points were calculated.

### Preparation of Preclinical Trial with Pig Samples

In vivo preclinical trials to evaluate the applicability of the sRFA‐needle were performed with pigs. The study was approved by the Institutional Animal Care and Use Committee, and all experiments were conducted in accordance with the institutional guidelines. To evaluate the ablation performance of the sRFA‐needle, RFA was performed using both the conventional RFA needle and the sRFA‐needle. The pigs were placed in a supine position, and a midline incision was made after sterile draping. One of the two radiologists performed the ablation procedures through the midline incision under ultrasonography guidance. During the RFA procedure, water coolant with ice was continuously circulated to the inner tube by a peristaltic pump to maintain the temperature of the needle body as cool as possible. Two to four RFA procedures were performed in each liver. After ablation, the livers were cut along the ablation surface, and the diameters of the ablation zones generated by the conventional and sRFA‐needles were compared. For the measurement, a source meter with two‐channel input (2602, Keithley, USA) was utilized, and the resistances of both T‐sensor and P‐sensor were continuously measured during the RFA procedures.

### Preparation of Clinical Trial

Clinical trials using the sRFA‐needles were performed for ten patients who were referred to the Department of Radiology at Samsung Medical Center for percutaneous RFA of hepatocellular carcinoma. The institutional review board approved this clinical trial, and informed consent was obtained from all patients (SMC 2019‐04‐088). The inclusion criteria for this clinical trial were as follows: 1) a single nodular HCC <3 cm; 2) absence of macrovascular invasion and extrahepatic metastasis during pretreatment imaging evaluation; 3) Child‐Pugh class A or B; 4) normal prothrombin time and platelet count >50 000 cells mL^−3^. The first ten patients who agreed to participate in the clinical trial were included. RFA was performed by one of five radiologists on duty. All procedures were performed under ultrasonography guidance, and, similar to the preclinical trial, water coolant with ice was continuously circulated to the inner tube to cool the needle body. All patients underwent contrast material‐enhanced multiphase liver CT immediately after RFA to evaluate technical success and assess immediate complications. After discharge, multiphase liver CT and laboratory tests, including tumor markers, were performed one month after the ablation to evaluate the efficacy of the technique. Technical success and technique efficacy one month after the procedure were both 100% (10/10). For the measurement of the sensor, commercial measurement equipment (PM‐1, RF Medical, Korea) was utilized considering the safety issue during the clinical study, which was certified by KTC. The equipment continuously measured both the minimum and maximum resistances of the T‐sensor and P‐sensor.

### Statistical Analysis

The ablated region after the RFA treatment in the preclinical trial was analyzed and quantified by image‐based analysis using the software ImageJ (National Institute of Health, U.S.). The major and minor axes of an ellipsoidal ablated region were estimated by taking the photograph of the cross‐sectioned porcine liver and analyzing the dimension in the image analysis software. In both the RFA process with the sRFA‐needle and the conventional RFA needle, four ablated tissues were utilized for further statistical analysis (i.e., *n* = 4). The mean value and standard deviation of those ablation regions were utilized in order to compare the ablation performance between the sRFA‐needle (experimental group) and a conventional RFA needle (control group).

## Conflict of Interest

The authors declare no conflict of interest.

## Supporting information

Supporting Information

Supplemental Video 1

## Data Availability

Research data are not shared.
