# Supplementary material for: Real‐Time Internal Steam Pop Detection during Radiofrequency Ablation with a Radiofrequency Ablation Needle Integrated with a Temperature and Pressure Sensor: Preclinical and Clinical Pilot Tests
Source: Adv Sci (Weinh). 2021 Aug 5;8(19):2100725. doi: 10.1002/advs.202100725 (PMC8498861; doi:10.1002/advs.202100725)
Supplement: Supplementary file 1 — Supporting Information [file ADVS-8-2100725-s001.pdf]

WILEY-VCH

© 2021 Wiley-VCH GmbH

ADVANCED SCIENCE
Open Access

Supporting Information

for *Adv. Sci.,* DOI: 10.1002/advs.202100725

Real-Time Internal Steam Pop Detection during Radiofrequency Ablation with a Radiofrequency Ablation Needle Integrated with a Temperature and Pressure Sensor: Pre-Clinical and Clinical Pilot Tests

Jaeho Park, Dong Ik Cha, Yongrok Jeong, Hayan Park, Jinwoo Lee, Tae Wook Kang, Hyo Keun Lim and Inkyu Park**

Supporting Information

Real-time Internal Steam Pop Detection during Radiofrequency Ablation with Temperature and Pressure Sensor Integrated Radiofrequency Ablation Needle: Pre-clinical and Clinical pilot test

*Jaeho Park[1,†], Dongik Cha[2,†], Yongrok Jeong[1,†], Hayan Park[2], Jinwoo Lee[3], Taewook Kang[2], Hyokeun Lim[2,4‡] and Inkyu Park[1‡]**

[1]Korea Advanced Institute of Science and Technology, Daejeon, South Korea, 34141

[2]Radiology and Center for Imaging Science, Samsung Medical Center, Sungkyunkwan University School of Medicine, Seoul, South Korea, 06351

[3]RF Medical Co. Ltd., Seoul, South Korea, 08511

[4]Department of Health Sciences and Technology, Samsung Advanced Institute for Health Sciences & Technology (SAIHST), Sungkyunkwan University School of Medicine, Seoul, SouthKorea, 06355

1. Design and details about the pressure chamber system for calibration of the sRFA-needle

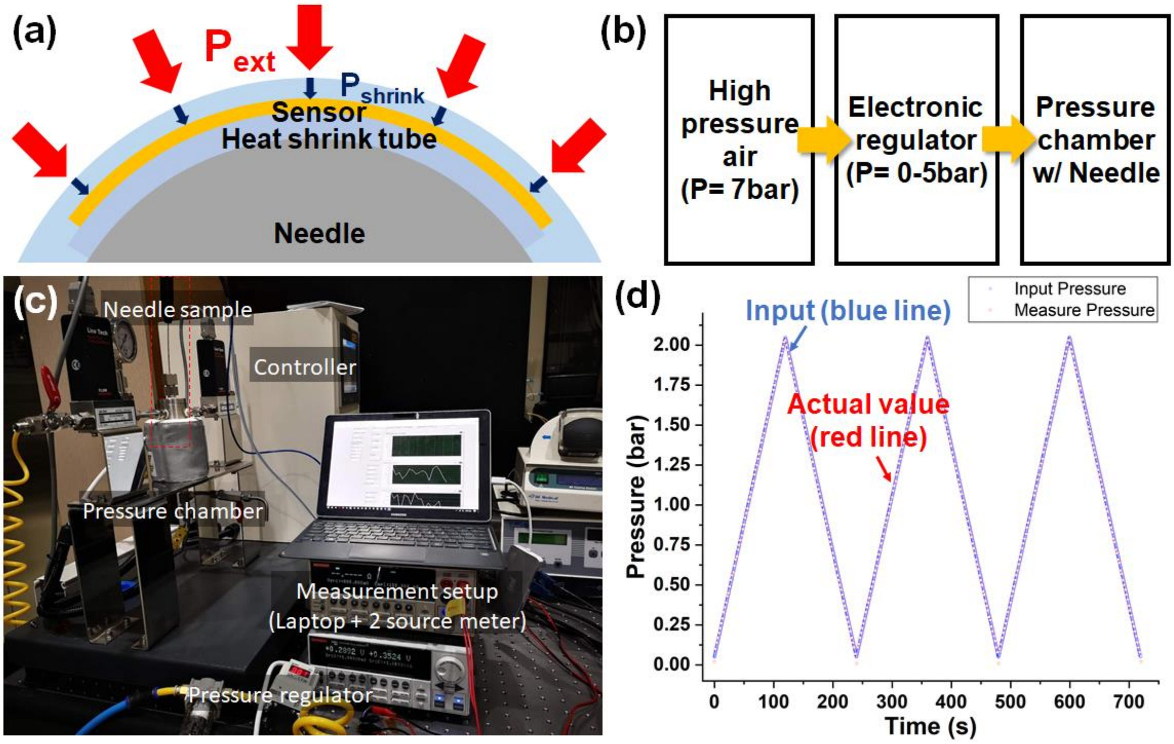

Figure S1. Details of calibration system of the pressure sensor on the RFA needle: (a) schematics for the cross-section of the fabricated system; (b) the schematical process of pressure chamber system; (c) experimental setup for the needle pressure calibration; (d) the graph for fast output pressure response in the chamber regulated by the electronic pressure regulator.

2. Working mechanism of the pressure sensor and its analysis based on the contact theory

From the contact theory[1], it is shown that the contact resistance can be calculated by the following equation:

$$\mathrm{R_c} = \left(\frac{\rho^2 \eta \pi H}{4F}\right)^{\frac{1}{2}} \qquad (1)$$

where ρ is the electrical resistivity, η is an empirical coefficient, H is the hardness of the material, and F is the load.

From this equation, and the previous paper of our group[2], the relationship between the applied pressure (P_t) on the sensor and the conductance of the sensor (G) can be concluded as follows:

$$\log P_t = a_1 + a_2 \log \frac{G}{G_0} \qquad (2)$$

where a_1 and a_2 are the calibration coefficients, and G_0 is the initial conductance.

As shown in **Figure S1(a)**, total pressure (P_t) applied on the sensor is the sum of external pressure (P) and the initial pressure by the heat shrink tube (P_s). Thus, equation (2) can be changed as follows:

$$\log P_t = \log(P + P_s) = a_1 + a_2 \log \frac{G}{G_0} \qquad (3)$$

In this equation (3), P and G/G_0 are variables, and others can be determined based on the experimental results. In manuscript, a_2 the value was expressed in a value. The above-mentioned behavior, which is a linear response between pressure and relative conductance in a log scale, could be found in the fabricated pressure sensor, and the change of contact resistance can be a main mechanism of the pr4ssure sensor.

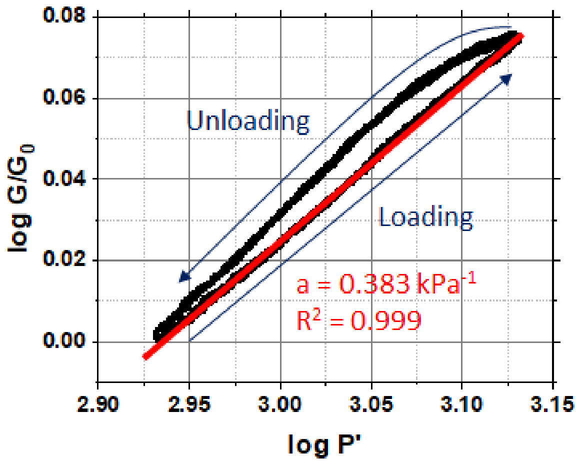

Figure S2. The response between pressure and relative conductance of the pressure sensor in a log scale.

3. Temperature effect on the pressure sensor and method for temperature compensation

Because the conductance of the sensor was affected by the temperature change, the temperature effect should be considered in the equation. The conductance of the sensor can be affected by temperature change in two points, briefly: the electron hopping nature of the carbon nanotube (CNT) [3,4] and the thermal expansion / shrinkage of the heat shrink tube. First, when it comes to the conductance change in CNT, since it follows the Arrhenius relationship, it can be expressed as follows:

$$G(T) = G_0 e^{-\frac{E_a}{RT}} = G(T_0)e^{-T_0/T} \leftrightarrow \log G(T) = \log G(T_0) - T_0 T^{-1} \qquad (4)$$

where T_0 is the reference temperature. Thus, in order to calibrate the change in conductance of CNT depends on the temperature, the inversely proportional term to the temperature should be inserted into the equation (3). And the result can be arranged as follows:

$$\log P_t = \mathrm{k}_1 + \mathrm{k}_2 \log G(T) + \mathrm{k}_3\, T^{-1} \qquad (5)$$

where k_1, k_2, and k_3 are the calibration coefficients.

Next, when it comes to the volume change of the heat shrink tube, the P_s term can be considered as the sum of initial pressure ($P_{s,i}$) and the pressure by the volume change of the heat shrink tube ($P_{s,t}$). Herein, because PET heat shrink tube is in the elastic deformation range (yield strength of PET: 48.9 MPa @ 27 °C, 38.6 MPa @ 100 °C [5]), $P_{s,t}$ is commensurate with the volume change ratio, that directly proportional to the temperature. Thus, this relationship can be applied in the equation as follows:

$$P_t = P + P_s = P + P_{s,i} + P_{s,t} = P + P_{s,i} + \mathrm{k}T \qquad (6)$$

where k is the calibration coefficient for the volume change induced by temperature change.

From equation (5) and (6), we can obtain the result as follows:

$$\log\ (P + P_{s,i} + \mathrm{k}\,T) = \mathrm{k}_1 + \mathrm{k}_2 \log G(T) + \mathrm{k}_3\, T^{-1} \qquad (7)$$

After some calculation, equation (7) can be changed as follows:

$$P = 10^{\mathrm{k}_1 + \mathrm{k}_2 \log G + \mathrm{k}_3\, T^{-1}} - P_{s,i} - \mathrm{k}T \qquad (8)$$

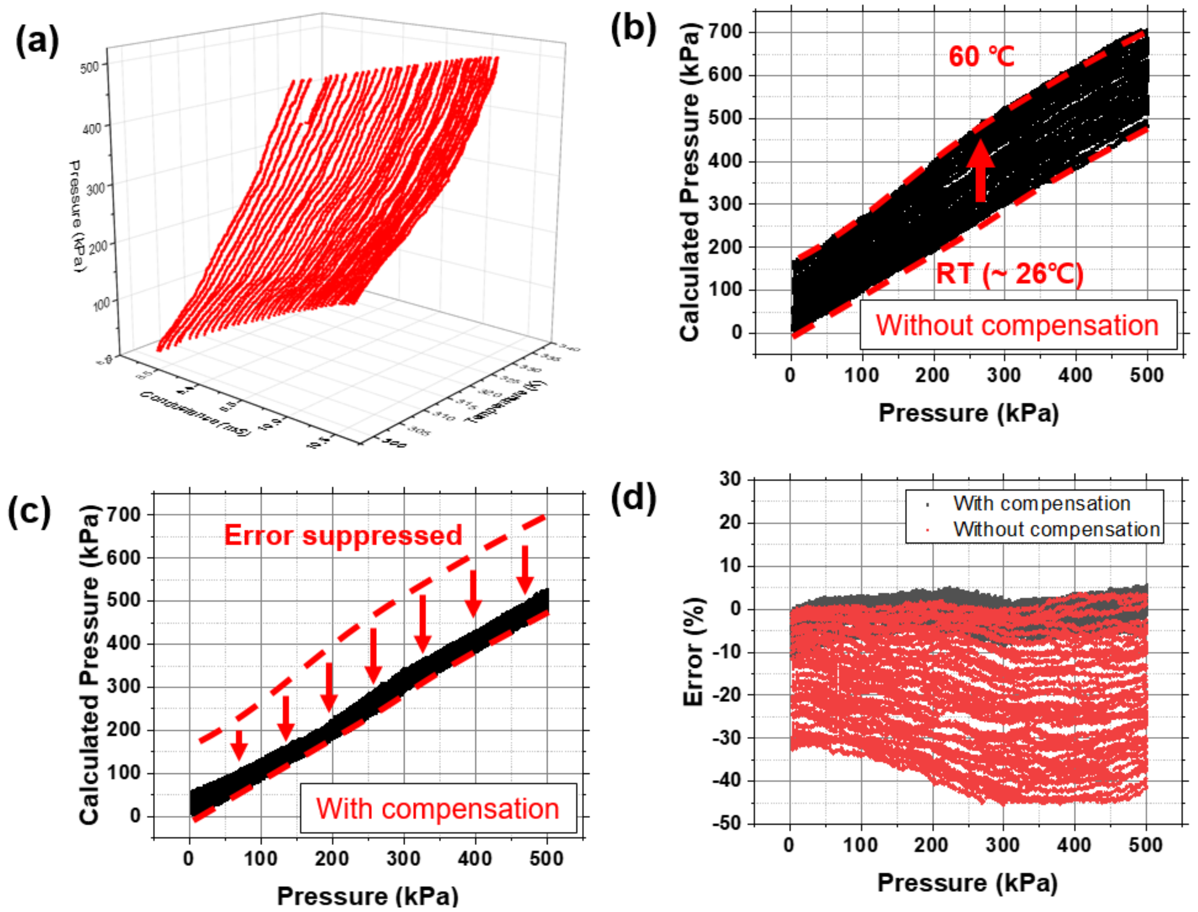

Figure S3. Experimental results of the temperature compensation: (a) Raw data of pressure response test with various temperature; (b) Results of calibration, without temperature compensation; (c) Results of calibration, with temperature compensation; (d) Error comparison between the calibration result without temperature compensation and that with temperature compensation.

4. Comparison of the ablation area between the conventional and the sRFA-needle

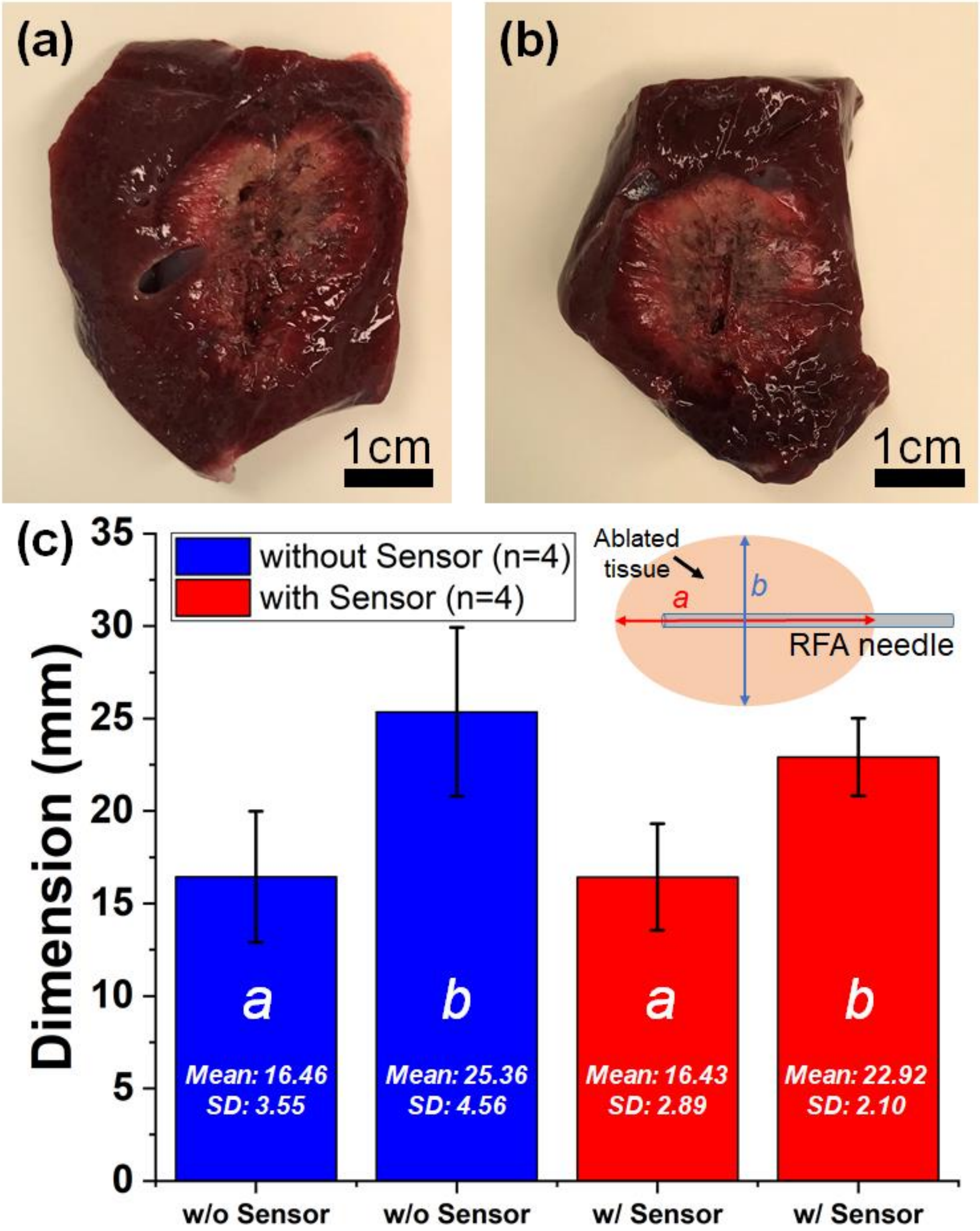

Figure S4. Comparison of the ablation performance between the conventional RFA needle and the sRFA-needle: photographic images of the excised tissue after the RFA procedure with (a) conventional RFA needle, and (b) the sRFA-needle; (c) the statistical analysis of the area of ablated tissue.

5. False-positive case of the sRFA-needle during the RFA procedure

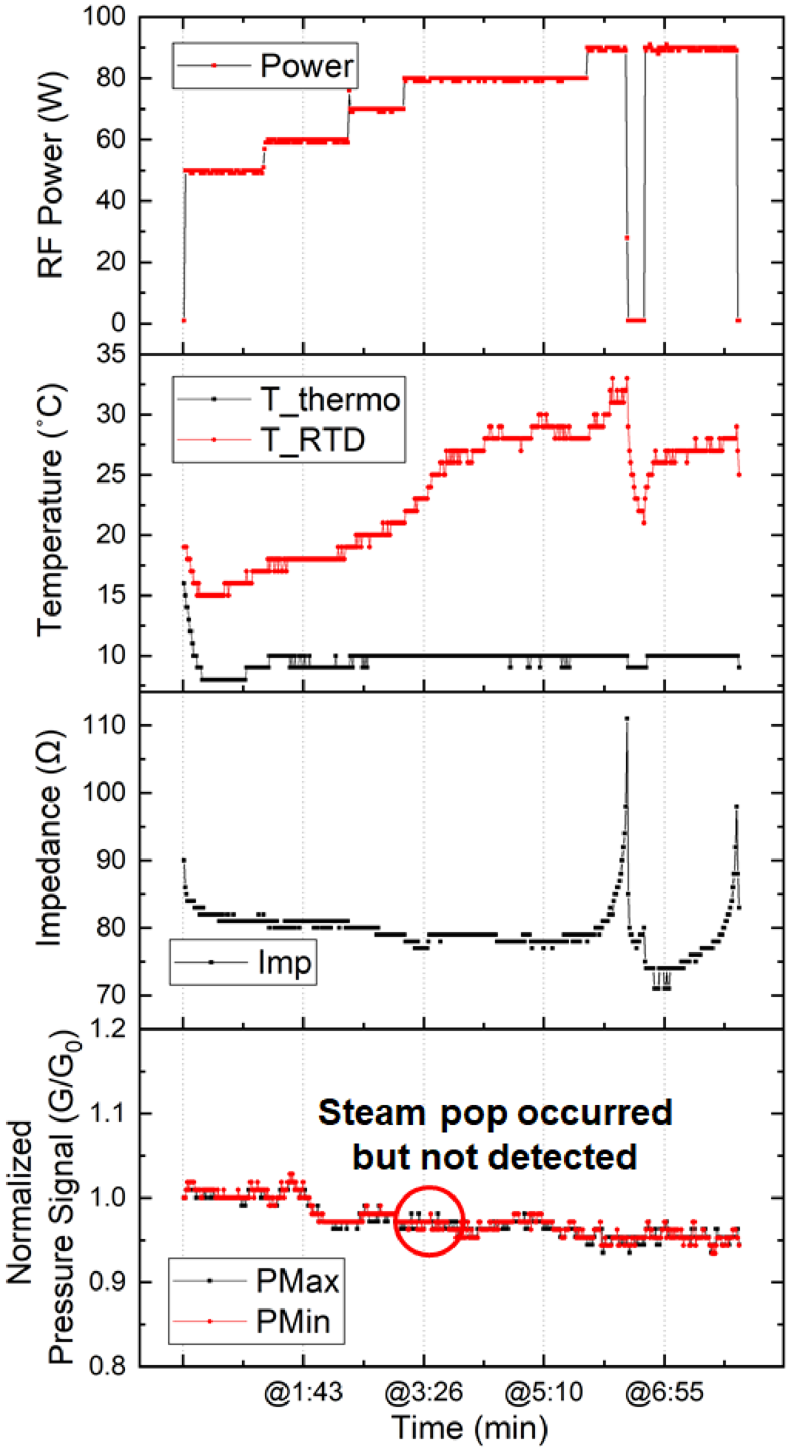

Figure S5. Graph of measured parameters during the RFA ablation procedure. There was a steam pop phenomena during the procdure, however, the sensor could not detect the occurance of the steam pop.

6. Summary of measurable parameters during clinical trials

Table S1. Summary of measurable parameters through the sRFA-needle (RF power, temperature, impedance, and relative pressure sensor signal) and their changes during clinical trials

**Figure name**	**Fig. 3(c)**	**Fig. 3(d)**	**Fig. 3(e)**
RF power (W)<br>(From start<br>to steam pop or roll-up)	100W 70s<br>➔ steam pop	50W 90s<br>➔ 60W 45s<br>➔ 70W 60s<br>➔ 80W 75s<br>➔ 90W 50s<br>➔ 100W 20s<br>➔ steam pop	30W 80s<br>➔ 40W 85s<br>➔ 50W 210s<br>➔ 60W 160s<br>➔ roll-up
Number of<br>steam pop	1	1	0
Temperature measured by<br>RTD at the surface of needle<br>(before/after steam pop) (°C)	25 ➔ 38<br>(ΔT= 13 °C)	24 ➔ 45<br>(ΔT= 21 °C)	n/a
Temperature measured by<br>thermocouple inside the needle<br>(before/after steam pop) (°C)	11 ➔ 12<br>(ΔT= 1 °C)	12 ➔ 12<br>(ΔT= 0 °C)	n/a
Electrical impedance measured<br>between the needle and the<br>ground pad<br>(before/after steam pop) (Ω)	78 ➔ 78<br>(ΔZ= 0 Ω)	66 ➔ 74<br>(ΔZ= 8 Ω)	n/a
Relative pressure sensor signal<br>@steam pop	0.990 ➔ 0.885<br>(ΔP_{rel}= 0.105)	0.982 ➔ 0.818<br>(ΔP_{rel}= 0.164)	n/a

7. The model for the temperature distribution analysis in the computational analysis

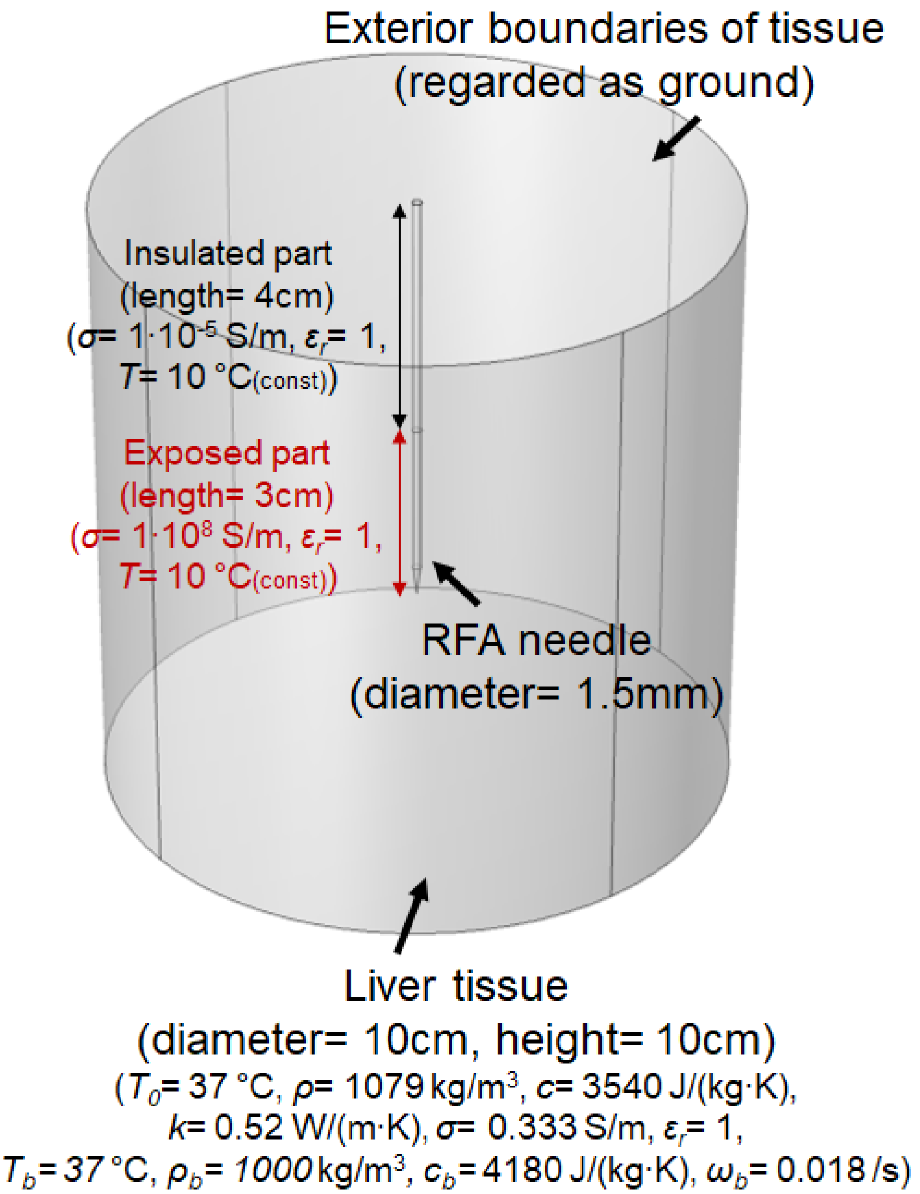

Figure S6. Overall model for computational analysis of the temperature distribution around the RFA needle. The parameters T, ρ, c, k, σ, ε_r are temperature, density, specific heat capacity, thermal conductivity, electrical conductivity, and relative permittivity, respectively. The subscript b denotes the parameters of blood flowing into the liver tissue.
